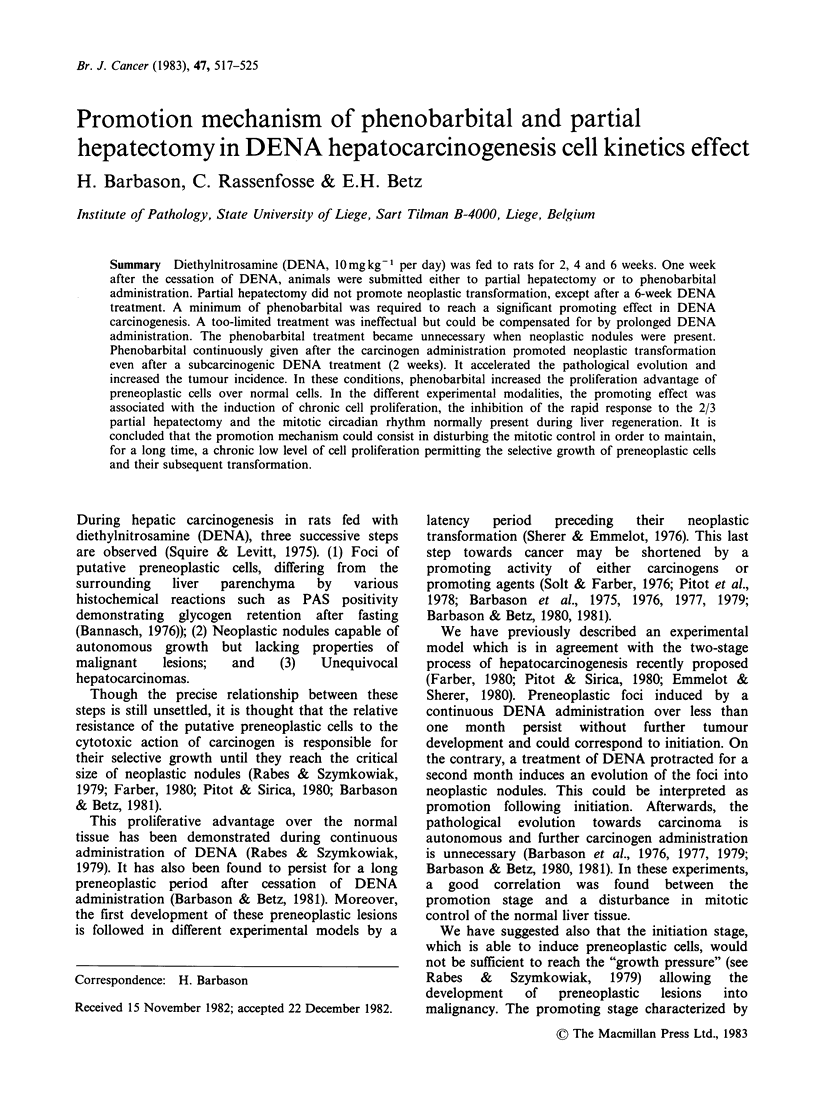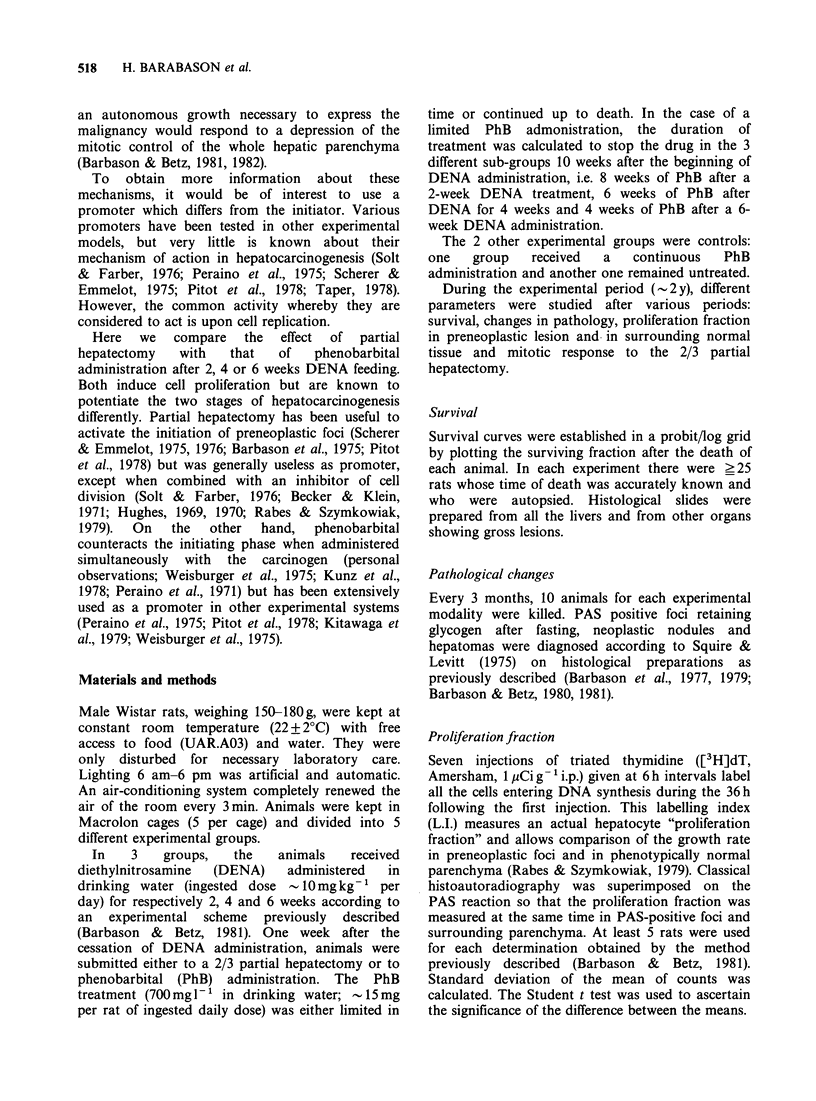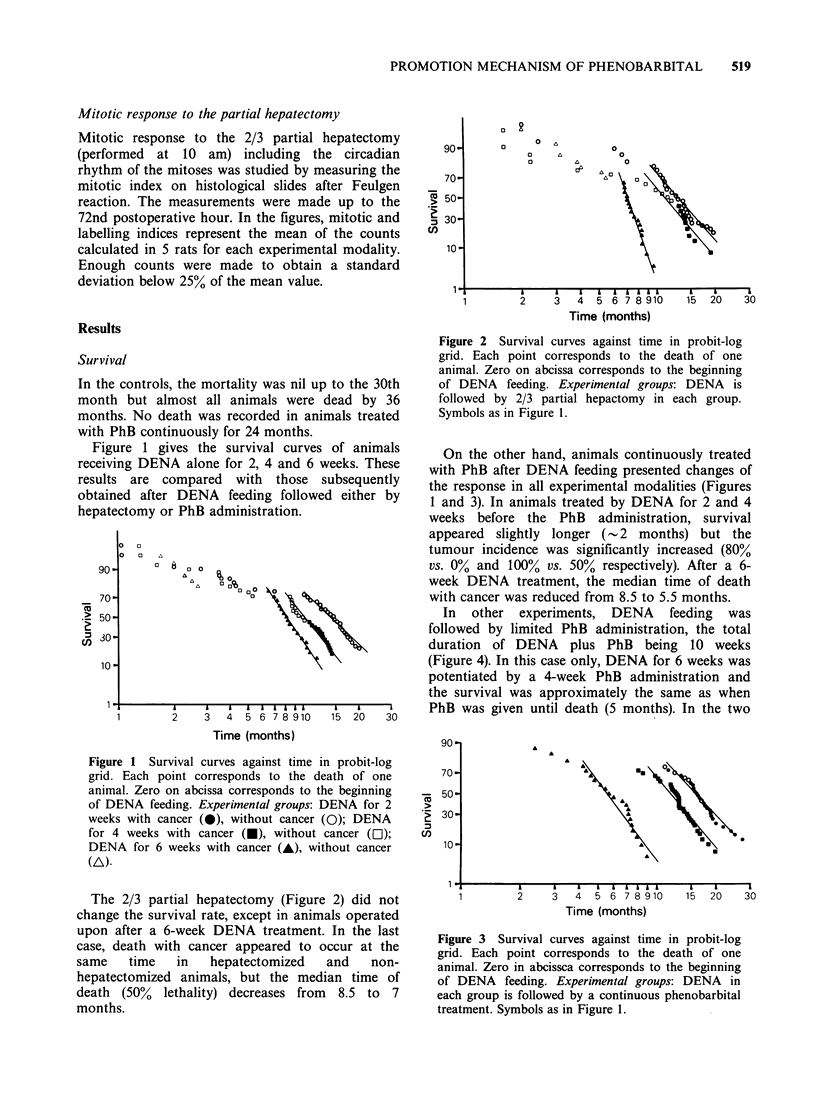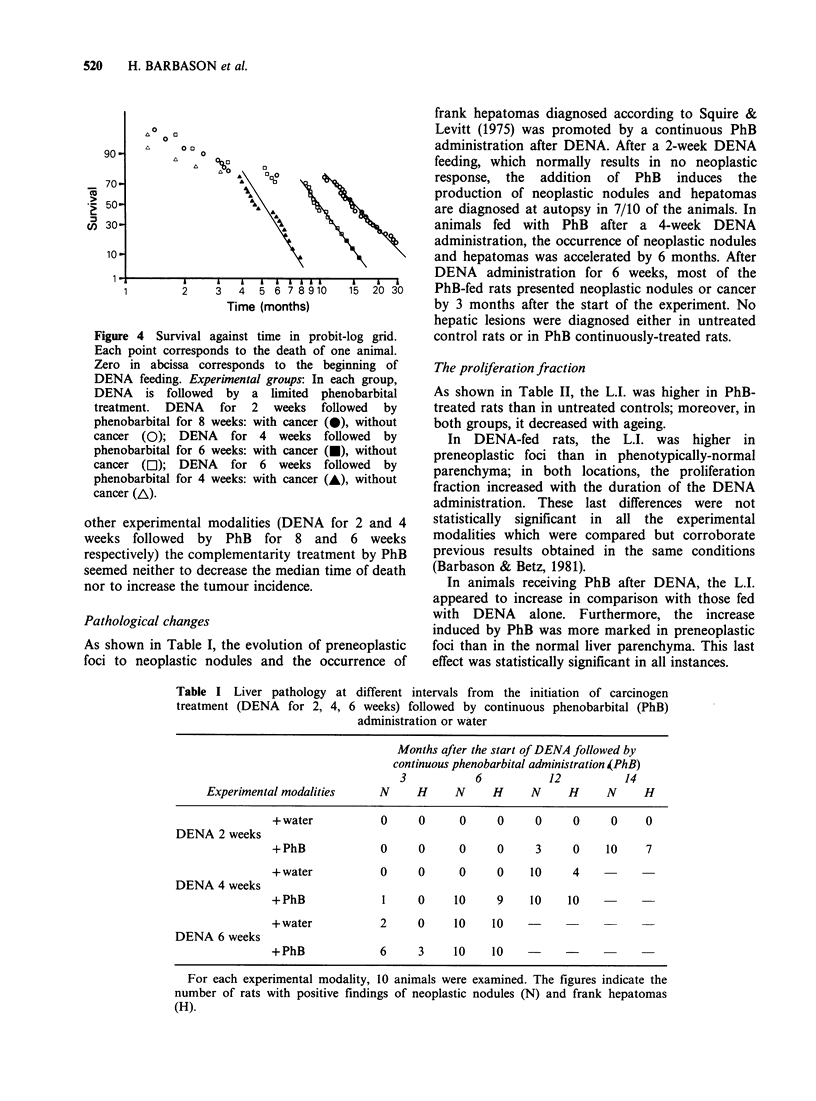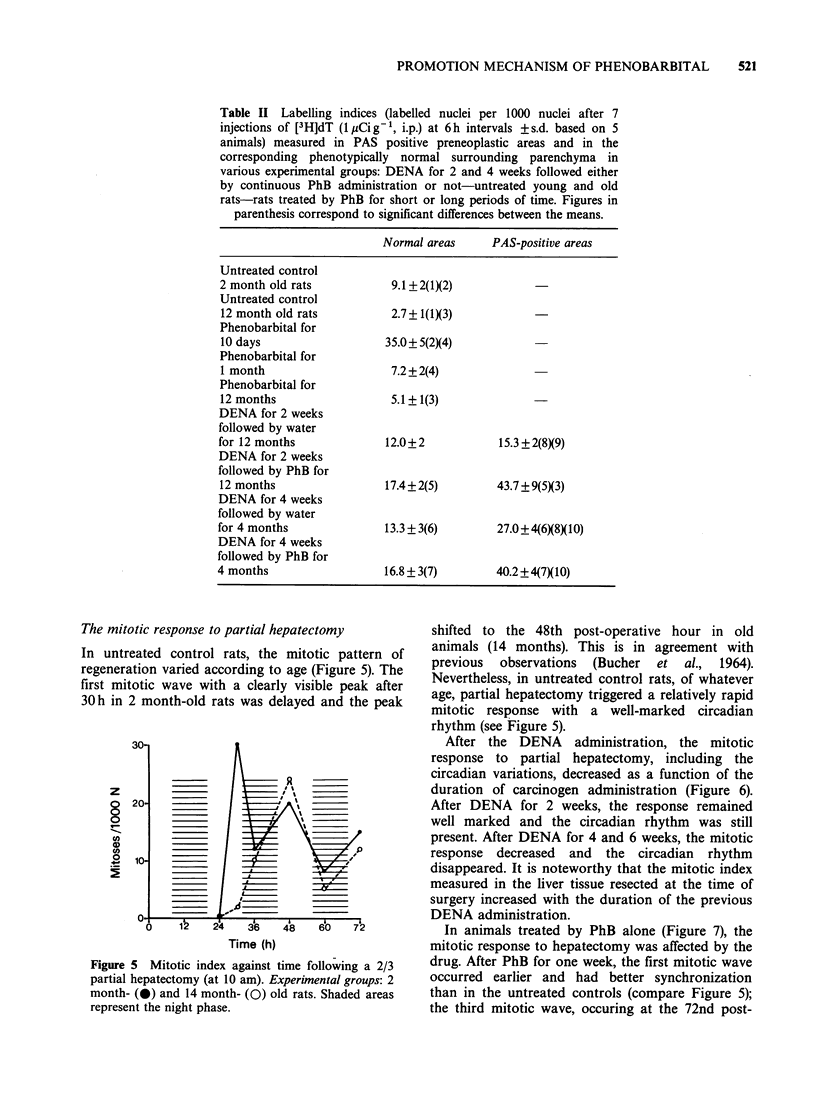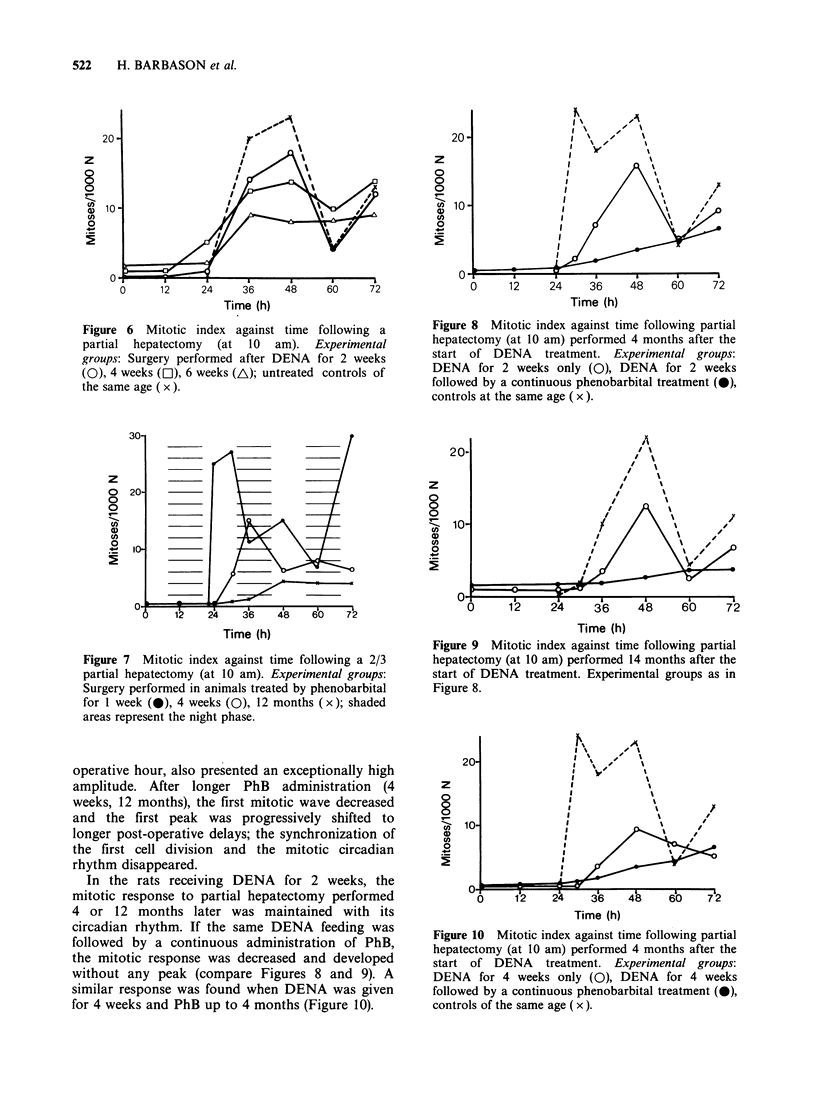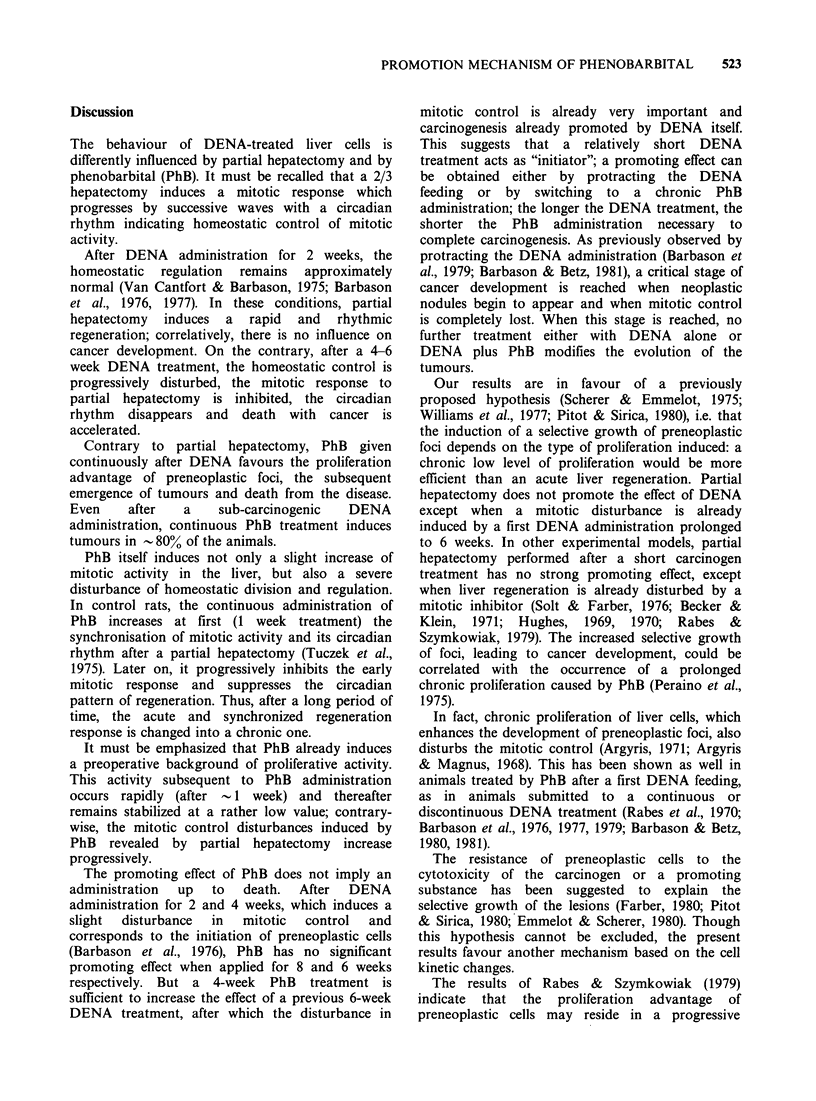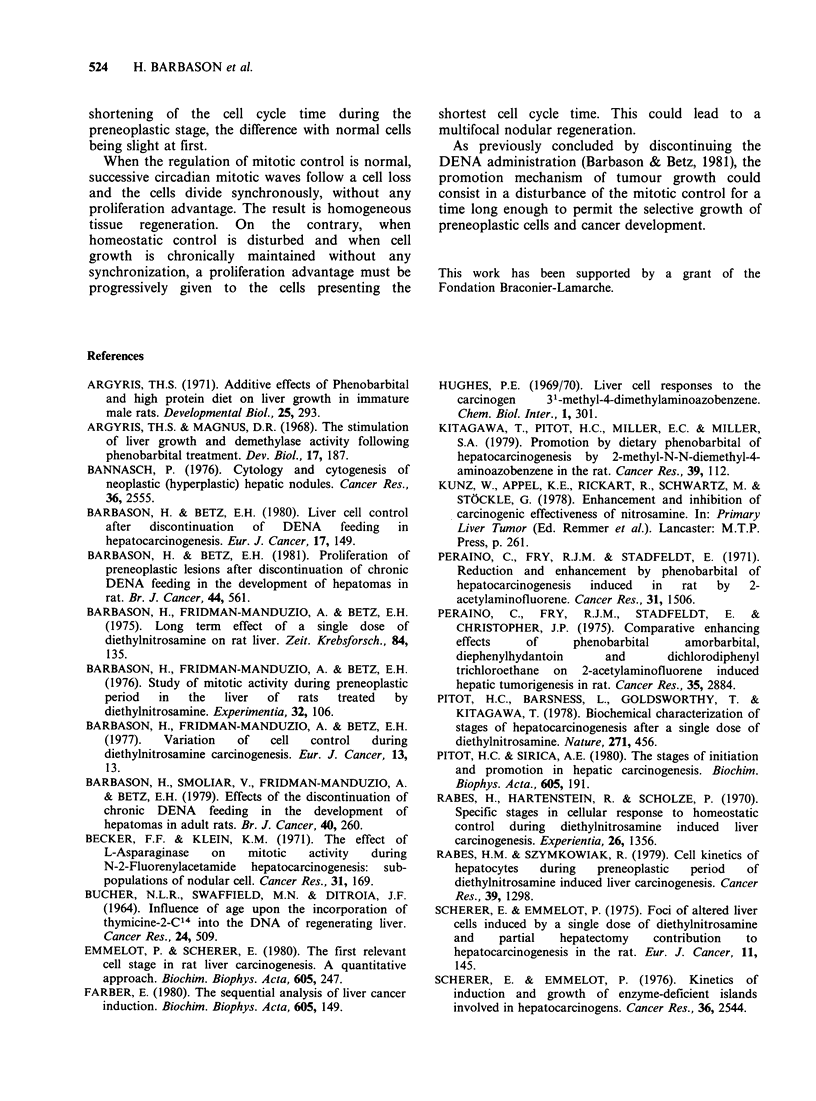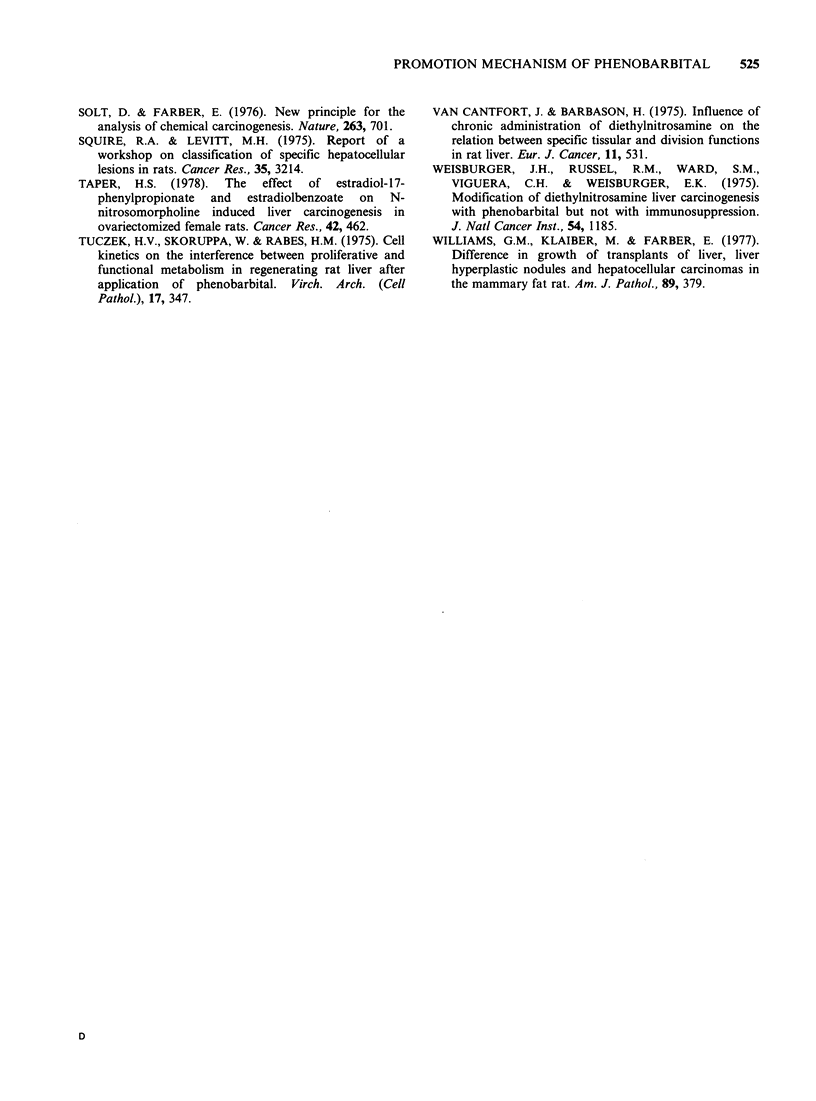# Promotion mechanism of phenobarbital and partial hepatectomy in DENA hepatocarcinogenesis cell kinetics effect.

**DOI:** 10.1038/bjc.1983.82

**Published:** 1983-04

**Authors:** H. Barbason, C. Rassenfosse, E. H. Betz

## Abstract

Diethylnitrosamine (DENA, 10 mg kg-1 per day) was fed to rats for 2, 4 and 6 weeks. One week after the cessation of DENA, animals were submitted either to partial hepatectomy or to phenobarbital administration. Partial hepatectomy did not promote neoplastic transformation, except after a 6-week DENA treatment. A minimum of phenobarbital was required to reach a significant promoting effect in DENA carcinogenesis. A too-limited treatment was ineffectual but could be compensated for by prolonged DENA administration. The phenobarbital treatment became unnecessary when neoplastic nodules were present. Phenobarbital continuously given after the carcinogen administration promoted neoplastic transformation even after a subcarcinogenic DENA treatment (2 weeks). It accelerated the pathological evolution and increased the tumour incidence. In these conditions, phenobarbital increased the proliferation advantage of preneoplastic cells over normal cells. In the different experimental modalities, the promoting effect was associated with the induction of chronic cell proliferation, the inhibition of the rapid response to the 2/3 partial hepatectomy and the mitotic circadian rhythm normally present during liver regeneration. It is concluded that the promotion mechanism could consist in disturbing the mitotic control in order to maintain, for a long time, a chronic low level of cell proliferation permitting the selective growth of preneoplastic cells and their subsequent transformation.


*Br. J. Cancer* (1983), **47**, 517–525

### Promotion mechanism of phenobarbital and partial hepatectomy in DENA hepatocarcinogenesis cell kinetics effect

H. Barbason, C. Rassenfosse & E.H. Betz

*Institute of Pathology, State University of Liege, Sart Tilman B-4000, Liege, Belgium*

**Summary** Diethylnitrosamine (DENA, $10\,mg\,kg^{-1}$ per day) was fed to rats for 2, 4 and 6 weeks. One week after the cessation of DENA, animals were submitted either to partial hepatectomy or to phenobarbital administration. Partial hepatectomy did not promote neoplastic transformation, except after a 6-week DENA treatment. A minimum of phenobarbital was required to reach a significant promoting effect in DENA carcinogenesis. A too-limited treatment was ineffectual but could be compensated for by prolonged DENA administration. The phenobarbital treatment became unnecessary when neoplastic nodules were present. Phenobarbital continuously given after the carcinogen administration promoted neoplastic transformation even after a subcarcinogenic DENA treatment (2 weeks). It accelerated the pathological evolution and increased the tumour incidence. In these conditions, phenobarbital increased the proliferation advantage of preneoplastic cells over normal cells. In the different experimental modalities, the promoting effect was associated with the induction of chronic cell proliferation, the inhibition of the rapid response to the 2/3 partial hepatectomy and the mitotic circadian rhythm normally present during liver regeneration. It is concluded that the promotion mechanism could consist in disturbing the mitotic control in order to maintain, for a long time, a chronic low level of cell proliferation permitting the selective growth of preneoplastic cells and their subsequent transformation.

During hepatic carcinogenesis in rats fed with diethylnitrosamine (DENA), three successive steps are observed (Squire & Levitt, 1975). (1) Foci of putative preneoplastic cells, differing from the surrounding liver parenchyma by various histochemical reactions such as PAS positivity demonstrating glycogen retention after fasting (Bannasch, 1976)); (2) Neoplastic nodules capable of autonomous growth but lacking properties of malignant lesions; and (3) Unequivocal hepatocarcinomas.

Though the precise relationship between these steps is still unsettled, it is thought that the relative resistance of the putative preneoplastic cells to the cytotoxic action of carcinogen is responsible for their selective growth until they reach the critical size of neoplastic nodules (Rabes & Szymkowiak, 1979; Farber, 1980; Pitot & Sirica, 1980; Barbason & Betz, 1981).

This proliferative advantage over the normal tissue has been demonstrated during continuous administration of DENA (Rabes & Szymkowiak, 1979). It has also been found to persist for a long preneoplastic period after cessation of DENA administration (Barbason & Betz, 1981). Moreover, the first development of these preneoplastic lesions is followed in different experimental models by a latency period preceding their neoplastic transformation (Sherer & Emmelot, 1976). This last step towards cancer may be shortened by a promoting activity of either carcinogens or promoting agents (Solt & Farber, 1976; Pitot *et al.*, 1978; Barbason *et al.*, 1975, 1976, 1977, 1979; Barbason & Betz, 1980, 1981).

We have previously described an experimental model which is in agreement with the two-stage process of hepatocarcinogenesis recently proposed (Farber, 1980; Pitot & Sirica, 1980; Emmelot & Sherer, 1980). Preneoplastic foci induced by a continuous DENA administration over less than one month persist without further tumour development and could correspond to initiation. On the contrary, a treatment of DENA protracted for a second month induces an evolution of the foci into neoplastic nodules. This could be interpreted as promotion following initiation. Afterwards, the pathological evolution towards carcinoma is autonomous and further carcinogen administration is unnecessary (Barbason *et al.*, 1976, 1977, 1979; Barbason & Betz, 1980, 1981). In these experiments, a good correlation was found between the promotion stage and a disturbance in mitotic control of the normal liver tissue.

We have suggested also that the initiation stage, which is able to induce preneoplastic cells, would not be sufficient to reach the "growth pressure" (see Rabes & Szymkowiak, 1979) allowing the development of preneoplastic lesions into malignancy. The promoting stage characterized by

Correspondence: H. Barbason

Received 15 November 1982; accepted 22 December 1982.

© The Macmillan Press Ltd., 1983

an autonomous growth necessary to express the malignancy would respond to a depression of the mitotic control of the whole hepatic parenchyma (Barbason & Betz, 1981, 1982).

To obtain more information about these mechanisms, it would be of interest to use a promoter which differs from the initiator. Various promoters have been tested in other experimental models, but very little is known about their mechanism of action in hepatocarcinogenesis (Solt & Farber, 1976; Peraino *et al.*, 1975; Scherer & Emmelot, 1975; Pitot *et al.*, 1978; Taper, 1978). However, the common activity whereby they are considered to act is upon cell replication.

Here we compare the effect of partial hepatectomy with that of phenobarbital administration after 2, 4 or 6 weeks DENA feeding. Both induce cell proliferation but are known to potentiate the two stages of hepatocarcinogenesis differently. Partial hepatectomy has been useful to activate the initiation of preneoplastic foci (Scherer & Emmelot, 1975, 1976; Barbason *et al.*, 1975; Pitot *et al.*, 1978) but was generally useless as promoter, except when combined with an inhibitor of cell division (Solt & Farber, 1976; Becker & Klein, 1971; Hughes, 1969, 1970; Rabes & Szymkowiak, 1979). On the other hand, phenobarbital counteracts the initiating phase when administered simultaneously with the carcinogen (personal observations; Weisburger *et al.*, 1975; Kunz *et al.*, 1978; Peraino *et al.*, 1971) but has been extensively used as a promoter in other experimental systems (Peraino *et al.*, 1975; Pitot *et al.*, 1978; Kitawaga *et al.*, 1979; Weisburger *et al.*, 1975).

### Materials and methods

Male Wistar rats, weighing 150–180 g, were kept at constant room temperature (22±2°C) with free access to food (UAR.A03) and water. They were only disturbed for necessary laboratory care. Lighting 6 am–6 pm was artificial and automatic. An air-conditioning system completely renewed the air of the room every 3 min. Animals were kept in Macrolon cages (5 per cage) and divided into 5 different experimental groups.

In 3 groups, the animals received diethylnitrosamine (DENA) administered in drinking water (ingested dose $\sim 10\,\mathrm{mg\,kg^{-1}}$ per day) for respectively 2, 4 and 6 weeks according to an experimental scheme previously described (Barbason & Betz, 1981). One week after the cessation of DENA administration, animals were submitted either to a 2/3 partial hepatectomy or to phenobarbital (PhB) administration. The PhB treatment ($700\,\mathrm{mg\,l^{-1}}$ in drinking water; $\sim 15$ mg per rat of ingested daily dose) was either limited in time or continued up to death. In the case of a limited PhB admonistration, the duration of treatment was calculated to stop the drug in the 3 different sub-groups 10 weeks after the beginning of DENA administration, i.e. 8 weeks of PhB after a 2-week DENA treatment, 6 weeks of PhB after DENA for 4 weeks and 4 weeks of PhB after a 6-week DENA administration.

The 2 other experimental groups were controls: one group received a continuous PhB administration and another one remained untreated.

During the experimental period ($\sim 2$ y), different parameters were studied after various periods: survival, changes in pathology, proliferation fraction in preneoplastic lesion and in surrounding normal tissue and mitotic response to the 2/3 partial hepatectomy.

### *Survival*

Survival curves were established in a probit/log grid by plotting the surviving fraction after the death of each animal. In each experiment there were $\geqq 25$ rats whose time of death was accurately known and who were autopsied. Histological slides were prepared from all the livers and from other organs showing gross lesions.

### *Pathological changes*

Every 3 months, 10 animals for each experimental modality were killed. PAS positive foci retaining glycogen after fasting, neoplastic nodules and hepatomas were diagnosed according to Squire & Levitt (1975) on histological preparations as previously described (Barbason *et al.*, 1977, 1979; Barbason & Betz, 1980, 1981).

### *Proliferation fraction*

Seven injections of triated thymidine ([$^3$H]dT, Amersham, $1\,\mu\mathrm{Ci\,g^{-1}}$ i.p.) given at 6 h intervals label all the cells entering DNA synthesis during the 36 h following the first injection. This labelling index (L.I.) measures an actual hepatocyte "proliferation fraction" and allows comparison of the growth rate in preneoplastic foci and in phenotypically normal parenchyma (Rabes & Szymkowiak, 1979). Classical histoautoradiography was superimposed on the PAS reaction so that the proliferation fraction was measured at the same time in PAS-positive foci and surrounding parenchyma. At least 5 rats were used for each determination obtained by the method previously described (Barbason & Betz, 1981). Standard deviation of the mean of counts was calculated. The Student $t$ test was used to ascertain the significance of the difference between the means.

*Mitotic response to the partial hepatectomy*

Mitotic response to the 2/3 partial hepatectomy (performed at 10 am) including the circadian rhythm of the mitoses was studied by measuring the mitotic index on histological slides after Feulgen reaction. The measurements were made up to the 72nd postoperative hour. In the figures, mitotic and labelling indices represent the mean of the counts calculated in 5 rats for each experimental modality. Enough counts were made to obtain a standard deviation below 25% of the mean value.

### Results

*Survival*

In the controls, the mortality was nil up to the 30th month but almost all animals were dead by 36 months. No death was recorded in animals treated with PhB continuously for 24 months.

Figure 1 gives the survival curves of animals receiving DENA alone for 2, 4 and 6 weeks. These results are compared with those subsequently obtained after DENA feeding followed either by hepatectomy or PhB administration.

**Figure 1** Survival curves against time in probit-log grid. Each point corresponds to the death of one animal. Zero on abcissa corresponds to the beginning of DENA feeding. *Experimental groups*: DENA for 2 weeks with cancer (●), without cancer (○); DENA for 4 weeks with cancer (■), without cancer (□); DENA for 6 weeks with cancer (▲), without cancer (△).

The 2/3 partial hepatectomy (Figure 2) did not change the survival rate, except in animals operated upon after a 6-week DENA treatment. In the last case, death with cancer appeared to occur at the same time in hepatectomized and non-hepatectomized animals, but the median time of death (50% lethality) decreases from 8.5 to 7 months.

**Figure 2** Survival curves against time in probit-log grid. Each point corresponds to the death of one animal. Zero on abcissa corresponds to the beginning of DENA feeding. *Experimental groups*: DENA is followed by 2/3 partial hepactomy in each group. Symbols as in Figure 1.

On the other hand, animals continuously treated with PhB after DENA feeding presented changes of the response in all experimental modalities (Figures 1 and 3). In animals treated by DENA for 2 and 4 weeks before the PhB administration, survival appeared slightly longer (~2 months) but the tumour incidence was significantly increased (80% *vs.* 0% and 100% *vs.* 50% respectively). After a 6-week DENA treatment, the median time of death with cancer was reduced from 8.5 to 5.5 months.

In other experiments, DENA feeding was followed by limited PhB administration, the total duration of DENA plus PhB being 10 weeks (Figure 4). In this case only, DENA for 6 weeks was potentiated by a 4-week PhB administration and the survival was approximately the same as when PhB was given until death (5 months). In the two

**Figure 3** Survival curves against time in probit-log grid. Each point corresponds to the death of one animal. Zero in abcissca corresponds to the beginning of DENA feeding. *Experimental groups*: DENA in each group is followed by a continuous phenobarbital treatment. Symbols as in Figure 1.

**Figure 4** Survival against time in probit-log grid. Each point corresponds to the death of one animal. Zero in abcissa corresponds to the beginning of DENA feeding. *Experimental groups*: In each group, DENA is followed by a limited phenobarbital treatment. DENA for 2 weeks followed by phenobarbital for 8 weeks: with cancer (●), without cancer (○); DENA for 4 weeks followed by phenobarbital for 6 weeks: with cancer (■), without cancer (□); DENA for 6 weeks followed by phenobarbital for 4 weeks: with cancer (▲), without cancer (△).

other experimental modalities (DENA for 2 and 4 weeks followed by PhB for 8 and 6 weeks respectively) the complementarity treatment by PhB seemed neither to decrease the median time of death nor to increase the tumour incidence.

*Pathological changes*

As shown in Table I, the evolution of preneoplastic foci to neoplastic nodules and the occurrence of frank hepatomas diagnosed according to Squire & Levitt (1975) was promoted by a continuous PhB administration after DENA. After a 2-week DENA feeding, which normally results in no neoplastic response, the addition of PhB induces the production of neoplastic nodules and hepatomas are diagnosed at autopsy in 7/10 of the animals. In animals fed with PhB after a 4-week DENA administration, the occurrence of neoplastic nodules and hepatomas was accelerated by 6 months. After DENA administration for 6 weeks, most of the PhB-fed rats presented neoplastic nodules or cancer by 3 months after the start of the experiment. No hepatic lesions were diagnosed either in untreated control rats or in PhB continuously-treated rats.

*The proliferation fraction*

As shown in Table II, the L.I. was higher in PhB-treated rats than in untreated controls; moreover, in both groups, it decreased with ageing.

In DENA-fed rats, the L.I. was higher in preneoplastic foci than in phenotypically-normal parenchyma; in both locations, the proliferation fraction increased with the duration of the DENA administration. These last differences were not statistically significant in all the experimental modalities which were compared but corroborate previous results obtained in the same conditions (Barbason & Betz, 1981).

In animals receiving PhB after DENA, the L.I. appeared to increase in comparison with those fed with DENA alone. Furthermore, the increase induced by PhB was more marked in preneoplastic foci than in the normal liver parenchyma. This last effect was statistically significant in all instances.

**Table I** Liver pathology at different intervals from the initiation of carcinogen treatment (DENA for 2, 4, 6 weeks) followed by continuous phenobarbital (PhB) administration or water

| Experimental modalities | | *Months after the start of DENA followed by continuous phenobarbital administration (PhB)* 3 | | 6 | | 12 | | 14 | |
|---|---|---|---|---|---|---|---|---|---|
| | | *N* | *H* | *N* | *H* | *N* | *H* | *N* | *H* |
| DENA 2 weeks | +water | 0 | 0 | 0 | 0 | 0 | 0 | 0 | 0 |
| | +PhB | 0 | 0 | 0 | 0 | 3 | 0 | 10 | 7 |
| DENA 4 weeks | +water | 0 | 0 | 0 | 0 | 10 | 4 | — | — |
| | +PhB | 1 | 0 | 10 | 9 | 10 | 10 | — | — |
| DENA 6 weeks | +water | 2 | 0 | 10 | 10 | — | — | — | — |
| | +PhB | 6 | 3 | 10 | 10 | — | — | — | — |

For each experimental modality, 10 animals were examined. The figures indicate the number of rats with positive findings of neoplastic nodules (N) and frank hepatomas (H).

**Table II** Labelling indices (labelled nuclei per 1000 nuclei after 7 injections of [$^3$H]dT (1 $\mu$Ci g$^{-1}$, i.p.) at 6 h intervals ±s.d. based on 5 animals) measured in PAS positive preneoplastic areas and in the corresponding phenotypically normal surrounding parenchyma in various experimental groups: DENA for 2 and 4 weeks followed either by continuous PhB administration or not—untreated young and old rats—rats treated by PhB for short or long periods of time. Figures in parenthesis correspond to significant differences between the means.

| | *Normal areas* | *PAS-positive areas* |
|---|---|---|
| Untreated control 2 month old rats | 9.1±2(1)(2) | — |
| Untreated control 12 month old rats | 2.7±1(1)(3) | — |
| Phenobarbital for 10 days | 35.0±5(2)(4) | — |
| Phenobarbital for 1 month | 7.2±2(4) | — |
| Phenobarbital for 12 months | 5.1±1(3) | — |
| DENA for 2 weeks followed by water for 12 months | 12.0±2 | 15.3±2(8)(9) |
| DENA for 2 weeks followed by PhB for 12 months | 17.4±2(5) | 43.7±9(5)(3) |
| DENA for 4 weeks followed by water for 4 months | 13.3±3(6) | 27.0±4(6)(8)(10) |
| DENA for 4 weeks followed by PhB for 4 months | 16.8±3(7) | 40.2±4(7)(10) |

*The mitotic response to partial hepatectomy*

In untreated control rats, the mitotic pattern of regeneration varied according to age (Figure 5). The first mitotic wave with a clearly visible peak after 30 h in 2 month-old rats was delayed and the peak shifted to the 48th post-operative hour in old animals (14 months). This is in agreement with previous observations (Bucher *et al.*, 1964). Nevertheless, in untreated control rats, of whatever age, partial hepatectomy triggered a relatively rapid mitotic response with a well-marked circadian rhythm (see Figure 5).

**Figure 5** Mitotic index against time following a 2/3 partial hepatectomy (at 10 am). *Experimental groups*: 2 month- (●) and 14 month- (○) old rats. Shaded areas represent the night phase.

After the DENA administration, the mitotic response to partial hepatectomy, including the circadian variations, decreased as a function of the duration of carcinogen administration (Figure 6). After DENA for 2 weeks, the response remained well marked and the circadian rhythm was still present. After DENA for 4 and 6 weeks, the mitotic response decreased and the circadian rhythm disappeared. It is noteworthy that the mitotic index measured in the liver tissue resected at the time of surgery increased with the duration of the previous DENA administration.

In animals treated by PhB alone (Figure 7), the mitotic response to hepatectomy was affected by the drug. After PhB for one week, the first mitotic wave occurred earlier and had better synchronization than in the untreated controls (compare Figure 5); the third mitotic wave, occuring at the 72nd post-

Figure 6 Mitotic index against time following a partial hepatectomy (at 10 am). *Experimental groups*: Surgery performed after DENA for 2 weeks (○), 4 weeks (□), 6 weeks (△); untreated controls of the same age (×).

Figure 7 Mitotic index against time following a 2/3 partial hepatectomy (at 10 am). *Experimental groups*: Surgery performed in animals treated by phenobarbital for 1 week (●), 4 weeks (○), 12 months (×); shaded areas represent the night phase.

operative hour, also presented an exceptionally high amplitude. After longer PhB administration (4 weeks, 12 months), the first mitotic wave decreased and the first peak was progressively shifted to longer post-operative delays; the synchronization of the first cell division and the mitotic circadian rhythm disappeared.

In the rats receiving DENA for 2 weeks, the mitotic response to partial hepatectomy performed 4 or 12 months later was maintained with its circadian rhythm. If the same DENA feeding was followed by a continuous administration of PhB, the mitotic response was decreased and developed without any peak (compare Figures 8 and 9). A similar response was found when DENA was given for 4 weeks and PhB up to 4 months (Figure 10).

Figure 8 Mitotic index against time following partial hepatectomy (at 10 am) performed 4 months after the start of DENA treatment. *Experimental groups*: DENA for 2 weeks only (○), DENA for 2 weeks followed by a continuous phenobarbital treatment (●), controls at the same age (×).

Figure 9 Mitotic index against time following partial hepatectomy (at 10 am) performed 14 months after the start of DENA treatment. Experimental groups as in Figure 8.

Figure 10 Mitotic index against time following partial hepatectomy (at 10 am) performed 4 months after the start of DENA treatment. *Experimental groups*: DENA for 4 weeks only (○), DENA for 4 weeks followed by a continuous phenobarbital treatment (●), controls of the same age (×).

**Discussion**

The behaviour of DENA-treated liver cells is differently influenced by partial hepatectomy and by phenobarbital (PhB). It must be recalled that a 2/3 hepatectomy induces a mitotic response which progresses by successive waves with a circadian rhythm indicating homeostatic control of mitotic activity.

After DENA administration for 2 weeks, the homeostatic regulation remains approximately normal (Van Cantfort & Barbason, 1975; Barbason *et al.*, 1976, 1977). In these conditions, partial hepatectomy induces a rapid and rhythmic regeneration; correlatively, there is no influence on cancer development. On the contrary, after a 4–6 week DENA treatment, the homeostatic control is progressively disturbed, the mitotic response to partial hepatectomy is inhibited, the circadian rhythm disappears and death with cancer is accelerated.

Contrary to partial hepatectomy, PhB given continuously after DENA favours the proliferation advantage of preneoplastic foci, the subsequent emergence of tumours and death from the disease. Even after a sub-carcinogenic DENA administration, continuous PhB treatment induces tumours in $\sim 80\%$ of the animals.

PhB itself induces not only a slight increase of mitotic activity in the liver, but also a severe disturbance of homeostatic division and regulation. In control rats, the continuous administration of PhB increases at first (1 week treatment) the synchronisation of mitotic activity and its circadian rhythm after a partial hepatectomy (Tuczek *et al.*, 1975). Later on, it progressively inhibits the early mitotic response and suppresses the circadian pattern of regeneration. Thus, after a long period of time, the acute and synchronized regeneration response is changed into a chronic one.

It must be emphasized that PhB already induces a preoperative background of proliferative activity. This activity subsequent to PhB administration occurs rapidly (after $\sim 1$ week) and thereafter remains stabilized at a rather low value; contrarywise, the mitotic control disturbances induced by PhB revealed by partial hepatectomy increase progressively.

The promoting effect of PhB does not imply an administration up to death. After DENA administration for 2 and 4 weeks, which induces a slight disturbance in mitotic control and corresponds to the initiation of preneoplastic cells (Barbason *et al.*, 1976), PhB has no significant promoting effect when applied for 8 and 6 weeks respectively. But a 4-week PhB treatment is sufficient to increase the effect of a previous 6-week DENA treatment, after which the disturbance in mitotic control is already very important and carcinogenesis already promoted by DENA itself. This suggests that a relatively short DENA treatment acts as "initiator"; a promoting effect can be obtained either by protracting the DENA feeding or by switching to a chronic PhB administration; the longer the DENA treatment, the shorter the PhB administration necessary to complete carcinogenesis. As previously observed by protracting the DENA administration (Barbason *et al.*, 1979; Barbason & Betz, 1981), a critical stage of cancer development is reached when neoplastic nodules begin to appear and when mitotic control is completely lost. When this stage is reached, no further treatment either with DENA alone or DENA plus PhB modifies the evolution of the tumours.

Our results are in favour of a previously proposed hypothesis (Scherer & Emmelot, 1975; Williams *et al.*, 1977; Pitot & Sirica, 1980), i.e. that the induction of a selective growth of preneoplastic foci depends on the type of proliferation induced: a chronic low level of proliferation would be more efficient than an acute liver regeneration. Partial hepatectomy does not promote the effect of DENA except when a mitotic disturbance is already induced by a first DENA administration prolonged to 6 weeks. In other experimental models, partial hepatectomy performed after a short carcinogen treatment has no strong promoting effect, except when liver regeneration is already disturbed by a mitotic inhibitor (Solt & Farber, 1976; Becker & Klein, 1971; Hughes, 1969, 1970; Rabes & Szymkowiak, 1979). The increased selective growth of foci, leading to cancer development, could be correlated with the occurrence of a prolonged chronic proliferation caused by PhB (Peraino *et al.*, 1975).

In fact, chronic proliferation of liver cells, which enhances the development of preneoplastic foci, also disturbs the mitotic control (Argyris, 1971; Argyris & Magnus, 1968). This has been shown as well in animals treated by PhB after a first DENA feeding, as in animals submitted to a continuous or discontinuous DENA treatment (Rabes *et al.*, 1970; Barbason *et al.*, 1976, 1977, 1979; Barbason & Betz, 1980, 1981).

The resistance of preneoplastic cells to the cytotoxicity of the carcinogen or a promoting substance has been suggested to explain the selective growth of the lesions (Farber, 1980; Pitot & Sirica, 1980; Emmelot & Scherer, 1980). Though this hypothesis cannot be excluded, the present results favour another mechanism based on the cell kinetic changes.

The results of Rabes & Szymkowiak (1979) indicate that the proliferation advantage of preneoplastic cells may reside in a progressive shortening of the cell cycle time during the preneoplastic stage, the difference with normal cells being slight at first.

When the regulation of mitotic control is normal, successive circadian mitotic waves follow a cell loss and the cells divide synchronously, without any proliferation advantage. The result is homogeneous tissue regeneration. On the contrary, when homeostatic control is disturbed and when cell growth is chronically maintained without any synchronization, a proliferation advantage must be progressively given to the cells presenting the shortest cell cycle time. This could lead to a multifocal nodular regeneration.

As previously concluded by discontinuing the DENA administration (Barbason & Betz, 1981), the promotion mechanism of tumour growth could consist in a disturbance of the mitotic control for a time long enough to permit the selective growth of preneoplastic cells and cancer development.

This work has been supported by a grant of the Fondation Braconier-Lamarche.

### References

ARGYRIS, TH.S. (1971). Additive effects of Phenobarbital and high protein diet on liver growth in immature male rats. *Developmental Biol.*, **25**, 293.

ARGYRIS, TH.S. & MAGNUS, D.R. (1968). The stimulation of liver growth and demethylase activity following phenobarbital treatment. *Dev. Biol.*, **17**, 187.

BANNASCH, P. (1976). Cytology and cytogenesis of neoplastic (hyperplastic) hepatic nodules. *Cancer Res.*, **36**, 2555.

BARBASON, H. & BETZ, E.H. (1980). Liver cell control after discontinuation of DENA feeding in hepatocarcinogenesis. *Eur. J. Cancer*, **17**, 149.

BARBASON, H. & BETZ, E.H. (1981). Proliferation of preneoplastic lesions after discontinuation of chronic DENA feeding in the development of hepatomas in rat. *Br. J. Cancer*, **44**, 561.

BARBASON, H., FRIDMAN-MANDUZIO, A. & BETZ, E.H. (1975). Long term effect of a single dose of diethylnitrosamine on rat liver. *Zeit. Krebsforsch.*, **84**, 135.

BARBASON, H., FRIDMAN-MANDUZIO, A. & BETZ, E.H. (1976). Study of mitotic activity during preneoplastic period in the liver of rats treated by diethylnitrosamine. *Experimentia*, **32**, 106.

BARBASON, H., FRIDMAN-MANDUZIO, A. & BETZ, E.H. (1977). Variation of cell control during diethylnitrosamine carcinogenesis. *Eur. J. Cancer*, **13**, 13.

BARBASON, H., SMOLIAR, V., FRIDMAN-MANDUZIO, A. & BETZ, E.H. (1979). Effects of the discontinuation of chronic DENA feeding in the development of hepatomas in adult rats. *Br. J. Cancer*, **40**, 260.

BECKER, F.F. & KLEIN, K.M. (1971). The effect of L-Asparaginase on mitotic activity during N-2-Fluorenylacetamide hepatocarcinogenesis: subpopulations of nodular cell. *Cancer Res.*, **31**, 169.

BUCHER, N.L.R., SWAFFIELD, M.N. & DITROIA, J.F. (1964). Influence of age upon the incorporation of thymicine-2-$C^{14}$ into the DNA of regenerating liver. *Cancer Res.*, **24**, 509.

EMMELOT, P. & SCHERER, E. (1980). The first relevant cell stage in rat liver carcinogenesis. A quantitative approach. *Biochim. Biophys. Acta*, **605**, 247.

FARBER, E. (1980). The sequential analysis of liver cancer induction. *Biochim. Biophys. Acta*, **605**, 149.

HUGHES, P.E. (1969/70). Liver cell responses to the carcinogen $3^1$-methyl-4-dimethylaminoazobenzene. *Chem. Biol. Inter.*, **1**, 301.

KITAGAWA, T., PITOT, H.C., MILLER, E.C. & MILLER, S.A. (1979). Promotion by dietary phenobarbital of hepatocarcinogenesis by 2-methyl-N-N-diemethyl-4-aminoazobenzene in the rat. *Cancer Res.*, **39**, 112.

KUNZ, W., APPEL, K.E., RICKART, R., SCHWARTZ, M. & STÖCKLE, G. (1978). Enhancement and inhibition of carcinogenic effectiveness of nitrosamine. In: *Primary Liver Tumor* (Ed. Remmer *et al.*). Lancaster: M.T.P. Press, p. 261.

PERAINO, C., FRY, R.J.M. & STADFELDT, E. (1971). Reduction and enhancement by phenobarbital of hepatocarcinogenesis induced in rat by 2-acetylaminofluorene. *Cancer Res.*, **31**, 1506.

PERAINO, C., FRY, R.J.M., STADFELDT, E. & CHRISTOPHER, J.P. (1975). Comparative enhancing effects of phenobarbital amorbarbital, diephenylhydantoin and dichlorodiphenyl trichloroethane on 2-acetylaminofluorene induced hepatic tumorigenesis in rat. *Cancer Res.*, **35**, 2884.

PITOT, H.C., BARSNESS, L., GOLDSWORTHY, T. & KITAGAWA, T. (1978). Biochemical characterization of stages of hepatocarcinogenesis after a single dose of diethylnitrosamine. *Nature*, **271**, 456.

PITOT, H.C. & SIRICA, A.E. (1980). The stages of initiation and promotion in hepatic carcinogenesis. *Biochim. Biophys. Acta.*, **605**, 191.

RABES, H., HARTENSTEIN, R. & SCHOLZE, P. (1970). Specific stages in cellular response to homeostatic control during diethylnitrosamine induced liver carcinogenesis. *Experientia*, **26**, 1356.

RABES, H.M. & SZYMKOWIAK, R. (1979). Cell kinetics of hepatocytes during preneoplastic period of diethylnitrosamine induced liver carcinogenesis. *Cancer Res.*, **39**, 1298.

SCHERER, E. & EMMELOT, P. (1975). Foci of altered liver cells induced by a single dose of diethylnitrosamine and partial hepatectomy contribution to hepatocarcinogenesis in the rat. *Eur. J. Cancer*, **11**, 145.

SCHERER, E. & EMMELOT, P. (1976). Kinetics of induction and growth of enzyme-deficient islands involved in hepatocarcinogens. *Cancer Res.*, **36**, 2544.

SOLT, D. & FARBER, E. (1976). New principle for the analysis of chemical carcinogenesis. *Nature*, **263**, 701.
SQUIRE, R.A. & LEVITT, M.H. (1975). Report of a workshop on classification of specific hepatocellular lesions in rats. *Cancer Res.*, **35**, 3214.
TAPER, H.S. (1978). The effect of estradiol-17-phenylpropionate and estradiolbenzoate on N-nitrosomorpholine induced liver carcinogenesis in ovariectomized female rats. *Cancer Res.*, **42**, 462.
TUCZEK, H.V., SKORUPPA, W. & RABES, H.M. (1975). Cell kinetics on the interference between proliferative and functional metabolism in regenerating rat liver after application of phenobarbital. *Virch. Arch. (Cell Pathol.)*, **17**, 347.
VAN CANTFORT, J. & BARBASON, H. (1975). Influence of chronic administration of diethylnitrosamine on the relation between specific tissular and division functions in rat liver. *Eur. J. Cancer*, **11**, 531.
WEISBURGER, J.H., RUSSEL, R.M., WARD, S.M., VIGUERA, C.H. & WEISBURGER, E.K. (1975). Modification of diethylnitrosamine liver carcinogenesis with phenobarbital but not with immunosuppression. *J. Natl Cancer Inst.*, **54**, 1185.
WILLIAMS, G.M., KLAIBER, M. & FARBER, E. (1977). Difference in growth of transplants of liver, liver hyperplastic nodules and hepatocellular carcinomas in the mammary fat rat. *Am. J. Pathol.*, **89**, 379.